# Surface Fractal Analysis for Estimating the Fracture Energy Absorption of Nanoparticle Reinforced Composites

**DOI:** 10.3390/ma5050922

**Published:** 2012-05-23

**Authors:** Brahmananda Pramanik, Tezeswi Tadepalli, P. Raju Mantena

**Affiliations:** Department of Mechanical Engineering, University of Mississippi, University, MS 38677, USA; E-Mails: tadepali@olemiss.edu (T.T.); meprm@olemiss.edu (P.R.M.)

**Keywords:** nanocomposites, low velocity punch-shear, fractured surface, digital microscopy, fractal dimension, fracture energy, fracture toughness

## Abstract

In this study, the fractal dimensions of failure surfaces of vinyl ester based nanocomposites are estimated using two classical methods, Vertical Section Method (VSM) and Slit Island Method (SIM), based on the processing of 3D digital microscopic images. Self-affine fractal geometry has been observed in the experimentally obtained failure surfaces of graphite platelet reinforced nanocomposites subjected to quasi-static uniaxial tensile and low velocity punch-shear loading. Fracture energy and fracture toughness are estimated analytically from the surface fractal dimensionality. Sensitivity studies show an exponential dependency of fracture energy and fracture toughness on the fractal dimensionality. Contribution of fracture energy to the total energy absorption of these nanoparticle reinforced composites is demonstrated. For the graphite platelet reinforced nanocomposites investigated, surface fractal analysis has depicted the probable ductile or brittle fracture propagation mechanism, depending upon the rate of loading.

## 1. Introduction

**Figure 1:**
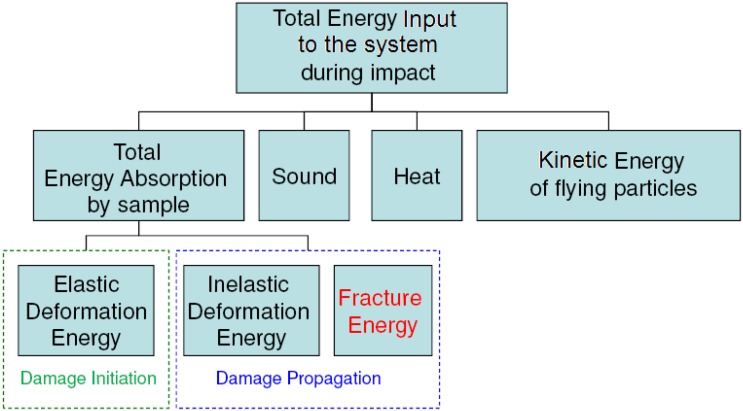
Contribution of fracture energy to the total energy absorption mechanism during low-velocity impact.

Nanocomposites are being considered for applications in marine structures subjected to blast waves and impact loading. Impact energy mitigation is a requirement for these structures. Characterizing energy absorption response of materials at higher strain rates has gained increasing attention from researchers. Figure 1 shows the fracture energy contribution as a part of the total absorbed energy during fracture propagation. It also indicates that a major portion of the total energy input to the system during loading is dissipated. Roughness of the fractured surface plays a major role in predicting fracture energy absorption.

Beginning with the pioneering work by Mandelbrot *et al.* [1], numerous investigators have focused on statistical characterization of the roughness of fractured surfaces [1,2,3,4,5,6,7,8,9,10]. It is now evident that the topography of crack surfaces can be described as being self-affine [10]. Self affinity is defined and well-described in the context of fractals by Mandelbrot [2]. As the crack propagates, the scale of tortuosity varies in different axes, which leads to fractal self-affinity of the fractured surface. Hence determining invariant fractal dimensionality of the surface becomes more tedious and multiple scaled-sampling dependent. Griffith states that brittle fracture occurs when the released strain energy is greater than the fracture energy required to create new fracture surfaces [11]. Consequently a more tortuous fractured surface indicates greater fracture energy absorption during crack propagation. 

**Figure 2:**
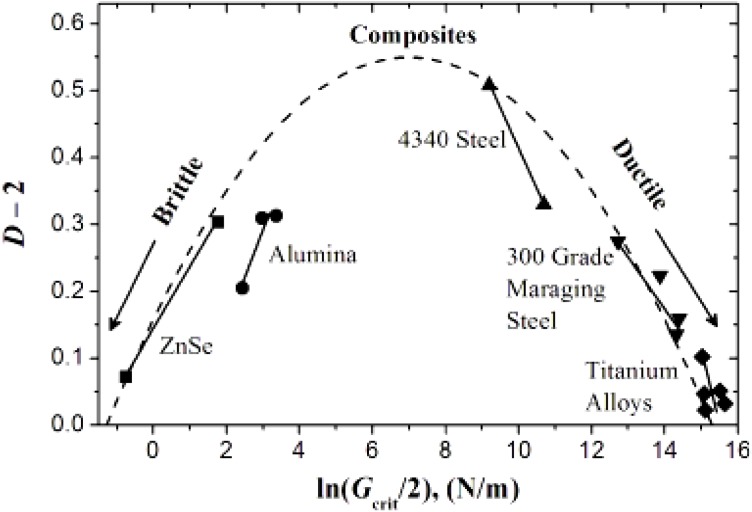
Relationship between fractal dimension of the fractured surface and fracture energy of different materials [9,12,13,14].

Cherepanov *et al.* [3] discussed several achievements in the field of fractal geometry that influenced fracture mechanics. Hotar *et al.* [4] applied fractal geometry in combination with statistical tools for the classification of surface roughness. Mecholosky [5] showed how fractal geometry can be used in estimating the theoretical strength of materials based on crack tip geometry and generated fracture surface. The applicability of the fractal concept within fracture mechanics has also been discussed in several publications. Kozlov *et al.* [6] showed that the fundamental concepts of fractal fracture mechanics are applicable to polymeric composites. Rodrigues *et al.* [7] applied the concept of fractals to explain the crack deflection toughening mechanism in ceramic materials. Ficker [8] derived the relationship between mechanical strength and fractal characteristics of porous gels and verified with experimental data. Williford [9] expressed the similarity relationship between fracture energy and surface roughness using fractal dimensionality. Lu *et al.* [9,12,13,14] extended Williford’s work to explain the uncertainties of predicting surface fractal dimensionality and illustrated (Figure 2) that the intuitive sense of tougher materials having rougher fracture surface is not commensurate with experimental observations. The relationships between roughness of fracture surfaces measured by fractal dimensions and toughness are exactly opposite for ductile and brittle materials. Composite materials are reported to have both possibilities.

Objective of the work presented here is to quantify fractal dimensionality of the failure surfaces for predicting the fracture energy and toughness of vinyl ester based nanocomposites. The methodologies of estimating fractal dimension of the fractured surfaces are based on the classical Vertical Section Method (VSM) and the Slit Island Method (SIM) described in literature [3]. For our work these conventional methodologies have been adapted such that a digital microscope may be utilized to capture the surface images, which are then digitally processed to create the roughness profiles required for VSM, and the 2D cross sections required for SIM. This eliminates specimen preparation difficulties and increases repeatability. Energy absorption due to fracture and toughness under quasi-static uni-axial tensile and low velocity punch-shear loading is estimated analytically from the surface fractal dimension. To verify the validity of these two procedures, conventional hot rolled A36 steel and grey cast iron specimens that fractured under quasi-static axial tensile loading are also analyzed. A study is conducted to quantify the sensitivity of both fracture energy and predicted fracture toughness to the fractal dimension of the failed surface profile. An investigation on the viscoelastic response of similar nanocomposites reported that the storage modulus varies with temperature as well as frequency in multi-frequency Dynamic Mechanical Analyzer (DMA) [15]. Frequency dependent storage modulus indicates the possibility of different failure characteristics of nanoparticle reinforced composites at varied loading rates. The application of fractal analysis has been considered in the current research to observe the post-failure characteristics of nanocomposite specimens subjected to two different loading rates, *i.e.*, quasi-static and low velocity punch-shear tests. The research findings reported in later sections demonstrate the applicability of surface fractal analysis for nanocomposites. 

## 2. Experimental Section 

### 2.1. Materials and Methods of Investigation

Pure Derakane 510A-40 brominated vinyl ester polymer is reinforced with 1.25 wt % xGnP (exfoliated graphite nanoplatelates) and 2.5 wt % xGnP in two different batches. Two batches are modified with a 10 wt % CTBN (Carboxy Terminated Butadiene Nitrile) rubbery toughening agent. The exfoliation and homogeneous dispersion of the nanoplatelets in polymer matrix are performed using sonication technique [16]. Coupons of these four different nanocomposite configurations are tested in quasi-static axial tensile and low velocity punch-shear loading according to ASTM standards D638 and D3763 respectively, and the improvement of mechanical properties is compared with respect to the pristine Derakane 510A-40 brominated vinyl ester polymer samples [17,18]. Specimens that are tested under low velocity punch-shear loading along with the corresponding material properties are shown in Table 1.

**Table 1:** **S**pecimens tested in low velocity punch-shear loading with the corresponding quasi-static uniaxial tensile properties used for estimating fracture energy and fracture toughness [17,18].

	Types of matrix	Type of reinforcement	Elastic modulus (GPa)	Ultimate strength (MPa)
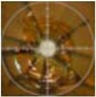	Brominated 510A-40 Vinyl ester	None	3.35	73.45
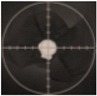	Brominated 510A-40 Vinyl ester	1.25 wt % xGnP	3.67	40.96
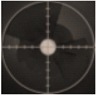	Brominated 510A-40 Vinyl ester	2.5 wt % xGnP	3.38	41.96
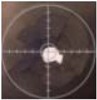	Brominated 510A-40 Vinyl ester	1.25 wt % xGnP + 10 wt % CTBN	3.48	44.58
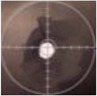	Brominated 510A-40 Vinyl ester	2.5 wt % xGnP + 10 wt % CTBN	4.68	27.68

### 2.2. Vertical Section Method

**Figure 3:**
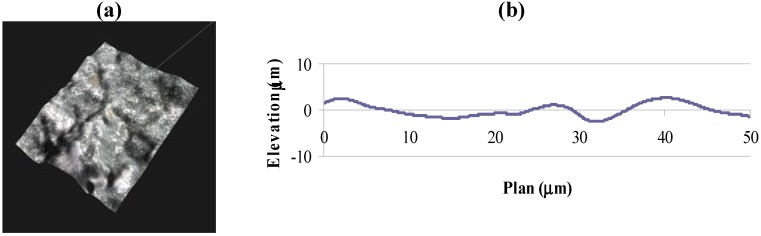
(**a**) 3D image and (**b**) vertical section profile of a typical fractured surface.

The fracture surfaces of failed specimens are studied with a Keyence VHX-600E digital microscope [19] which generates a series of 3D-images (Figure 3a) at optical magnifications of 500×, and 1000× to 5000× with 1000× increment. Global slope of the cracked surface may also contribute to the variation in computed fractal dimension. At each magnification level, the profile roughness (Figure 3b) is estimated by the ratio (*R_L_*) of vertical section profile length (obtained from 3D Profile Measurement Software, VHX-H2MK Ver 1.1) to a fixed projection length of 50 μm, consistent for all cases. Surface roughness (*R_S_*) is calculated using the following expression [3]:

(1) $$R_{s}=\frac{4}{\pi }\left(R_{L}-1\right)+1$$

**Figure 4:**
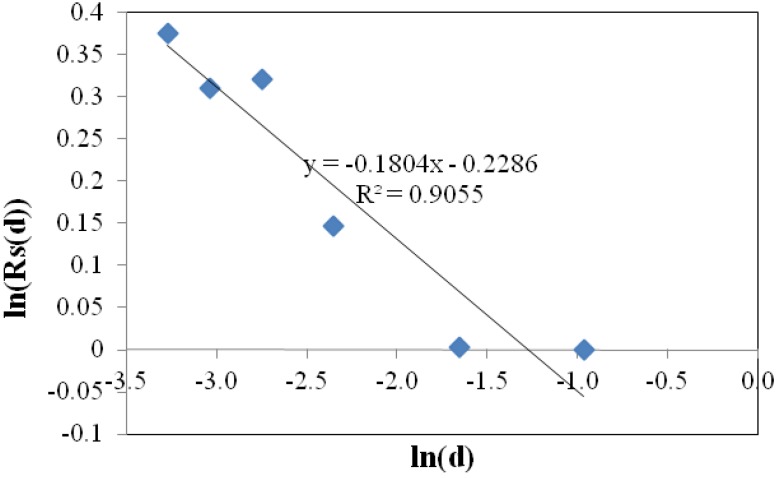
Typical fraction of surface fractal dimension from the slope of regression line in Vertical Section Method (VSM).

The calibration factor, which is the constant horizontal distance between two consecutive data points on the vertical section profile at a specific magnification level, is used as measurement scale (*d*). The surface roughness (*R_S_*) is related to surface fractal dimensionality (*D_S_*) [3] as:

(2) $$R_{s}\left(d\right)=Kd^{D_{S}-2}$$

where *K* is the model constant. The fractional part of *D_S_* is evaluated from the slope of linear regression of *R_S_* versus *d*, plotted on log-log scale (Figure 4).

### 2.3. Slit Island Method

**Figure 5:**
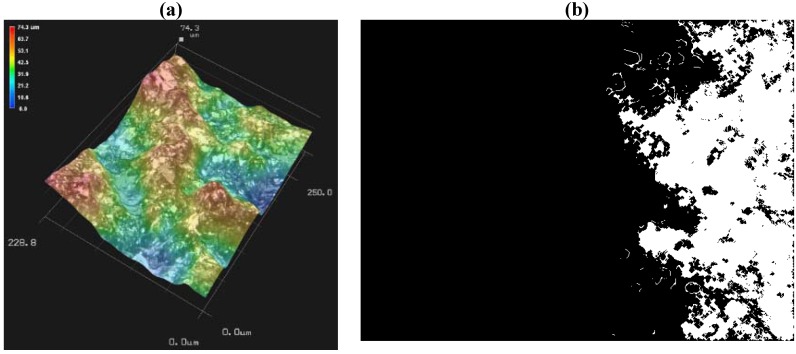
(**a**) 3D topography of a typical fractured surface; and (**b**) Black-and-White (BW) 2D summed-image (1600 × 1200 pixels in 250 µm × 228.8 µm viewport) at 10 µm depth representing islands.

The evident dependency of dimensionality on the ratio of area (*S*) and perimeter (*P*) is applied to estimate the fractional part of *D_S_*. Multiple fractured surface 2D images are captured at 1000× magnification by varying the depth of the focal plane of the microscope in 1 micron increment up to a depth of 10 µm under ambient light source. These images represent the closed-loop contour lines at various focal depths. These images are converted to grayscale image for applying “Sobel” edge detection method which returns Black-and-White (BW) images with edges computed based on maximum image intensity gradient. A MATLAB^®^ code, developed in-house, was used for performing image-summation of ten depth-wise consecutive images (Figure 5a) to obtain slit islands at an effective depth of 10 µm along with respective area and perimeter estimation. The image processing functions available in MATLAB^®^ are applied to reduce noise. The white colored area in Figure 5b is considered to be the “Slit Island”. The boundary of each island is plotted with a least-count that is one pixel long (0.16 µm), which is considered here as the ‘yardstick’ for the Richardson approach in estimating dimensionality of the fractured surface. The area of each island is obtained from the region within this boundary.

**Figure 6:**
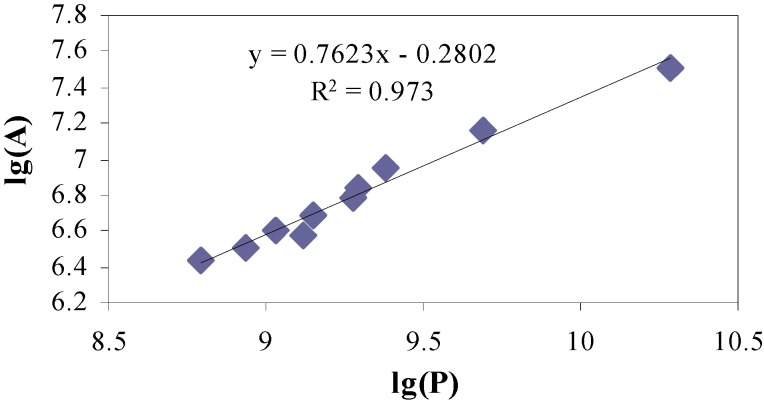
Typical surface fractal dimension estimation from the slope of the regression line in Slit Island Method (SIM).

The coordinate of each vertex on the boundary line is determined and the perimeter of each island is estimated by the cumulative summation of the distances of two consecutive vertices. The area and perimeter of all islands are summed to obtain the corresponding total area (*S*) and perimeter (*P*) of fractal islands. The respective *S* and *P* are estimated using ten different ‘yardsticks’ of consecutively increasing length. The least-square fit line is drawn in spread sheet application through *S* versus *P* plotted on log-log scale (Figure 6). Slope of this line indicates the fractional part of the fractal dimension (*D_S_*) for each crack surface [3].

### 2.4. Fracture Energy and Toughness Estimation

The fractal dimension (*D_S_*) of the fracture surface described in the previous section, is used to determine fracture energy (*J_IC_*) using the following Equations [20]:

(3) $$J_{IC}=CL^{\left(D_{S}-2\right)}$$

where,

(4) $$C=\frac{\pi {S_{Y}}^{2}}{E}$$

*J_IC_* = fracture energy, *L* = scale of observation (approximated by consistent dimensionality), *S_Y_* = Yield strength (ultimate strength for brittle materials), *E* = Young’s modulus. From the estimated fracture energy (*J_IC_*), the toughness (*K_IC_*) was evaluated using the following Equation [20]:

(5) $$K_{IC}=\sqrt{J_{IC}E}$$

**Figure 7:**
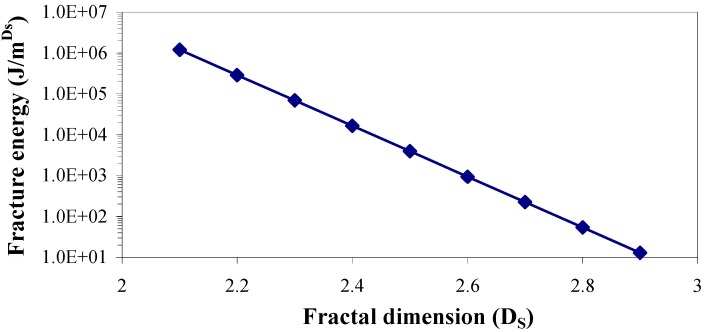
Theoretical relation of fracture energy with fractal dimension in parametric sensitivity study.

Parametric sensitivity of the fracture energy obtained from the fractal dimension has been investigated using the well established Equations 3 and 4. Results plotted in Figure 7 show that fracture energy decreases exponentially with linear increment of fractal dimension, indicating that the fracture energy is highly sensitive to the magnitude of the fractal dimension.

## 3. Results and Discussion

A series of experiments to determine fractal dimensionality of the nanoreinforced fractured surface are organized in two sections. The first section validates the applicability of the two methods to our investigation. VSM and SIM are used independently to determine the fractal dimension of conventional hot rolled steel and cast iron fractured under quasi-static axial tensile tests. Dimensionalities so obtained are compared with the trend of tortuosity evaluated using the concept explained in earlier studies [11,17]. A large difference is observed between the dimensionalities obtained from VSM and SIM. The possibility for systematic errors is higher in Vertical Section Method, which is based on surface fractal measurements covering a broad range of scales with limited steps of magnification. However, such errors are reduced in the case of Slit Island Method, but at the expense of introducing scale dependent estimates since the fractal dimensionality is estimated at a certain scale. The dimensionality values of cast iron and steel obtained by VSM method are found to be similar to values published in literatures [21,22], and follow a trend similar to that observed for tortuosity. Hence analysis for fracture energy and fracture toughness of all specimens in current investigation is based on the dimensionalities obtained from VSM. The second section determines the fractal dimensionality of nanoparticle reinforced composite specimens fractured under quasi-static tensile and under low velocity punch-shear loading. Average of fractal dimensionalities obtained from five locations on the fractured surface of each specimen is estimated for further analysis. The respective average fractal dimensionalities are shown in Table 2. In general, a higher dimensionality is observed in dynamically punch-sheared specimens as compared to specimens that failed under quasi-static axial tension. 

**Table 2:** Surface fractal dimension of nanocomposites under quasi-static axial tensile and low velocity punch-shear loading.

	Quasi-static axial tensile test			Low velocity punch-shear test		
	VSM *	SIM *	Tortuosity ^#^	VSM *	SIM *	Tortuosity ^#^
A36 hot rolled Steel [18,19]	0.078	0.700	0.037	–	–	–
Cast Iron	0.159	0.865	0.085	–	–	–
Vinyl ester	0.139	0.348	0.026	0.181	0.742	0.042
Vinyl ester + 1.25 wt % xGnP	0.161	0.463	0.034	0.269	0.766	0.075
Vinyl ester + 2.5 wt % xGnP	0.189	0.662	0.039	0.127	0.647	0.051
Vinyl ester + 1.25 wt % xGnP + 10 wt % CTBN	0.182	0.574	0.042	0.161	0.743	0.063
Vinyl ester + 2.5 wt % xGnP + 10 wt % CTBN	0.214	0.622	0.056	0.127	0.703	0.050

* Fractional part of fractal dimensions; ^#^ Surface roughness [11,17].

**Figure 8:**
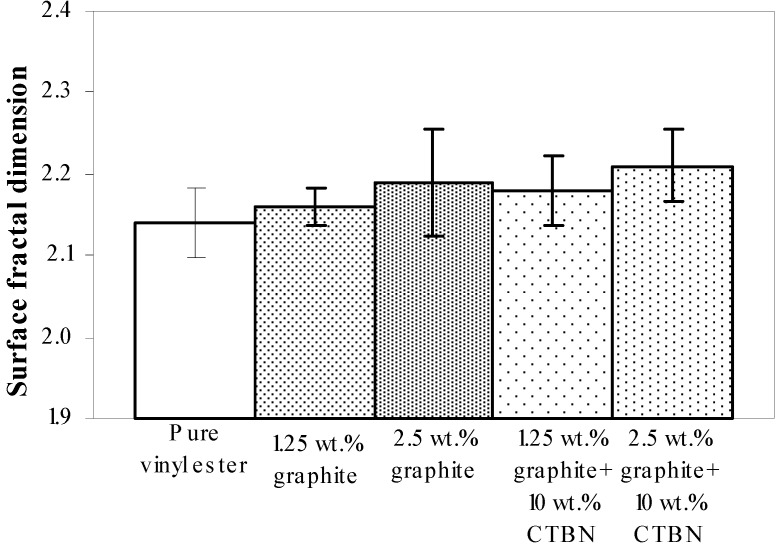
Surface fractal dimension (from VSM) of nanocomposites under quasi-static axial tensile loading.

**Figure 9:**
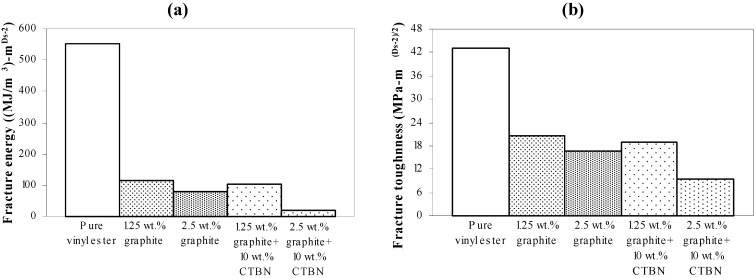
Theoretically estimated (**a**) fracture energy and (**b**) fracture toughness of nanocomposites under quasi-static axial tensile loading.

Experimental results obtained using VSM are further analyzed to study the correlation of surface fractal dimension (Figure 8) with fracture energy, as well as fracture toughness, under both quasi-static tensile and low velocity punch-shear loading. Nanocomposite specimens that fractured under quasi-static tensile loading show an increase in the fracture surface dimensionality with increasing graphite and CTBN reinforcement (Figure 8). Fracture energy and the corresponding fracture toughness (estimated from average fractal dimensionality using theoretical Equations 3 to 5, and shown in Figure 9 (a,b) respectively) demonstrate a decrease in energy absorption due to fracture surface creation with increase in nanoparticle reinforcement. 

**Figure 10:**
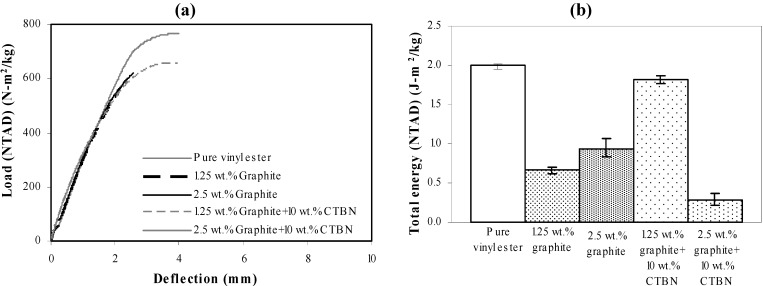
Experimentally obtained (**a**) load-deflection response; and (**b**) total energy (NTAD) absorption of nanocomposites under quasi-static axial tensile loading.

Area under the experimental load-deflection response curve (Figure 10a) provides the total energy absorption corresponding to all failure mechanisms (Figure 10b) under quasi-static tensile loading. It is to be noted that all experimental data has been normalized to the respective specimen areal density-NTAD [17,18]. 

Though fracture contributes to only a part of this total energy absorption, overall trend between the theoretically estimated fracture energy and experimentally obtained total energy absorption are not in good agreement for quasi-static axial tensile loading.

**Figure 11:**
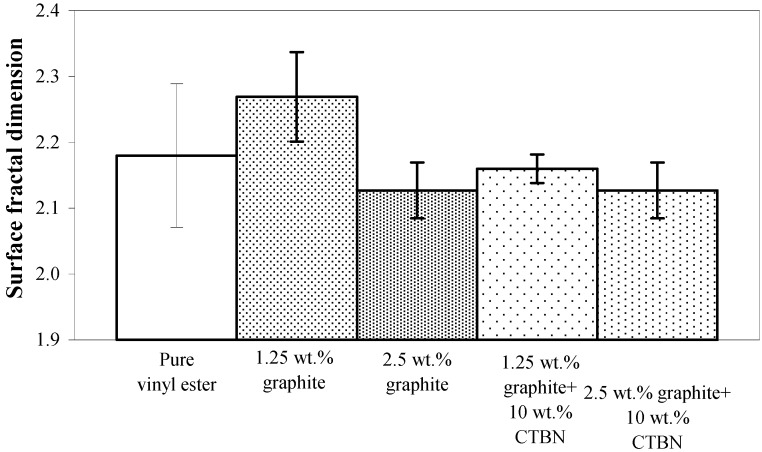
Surface fractal dimension (from VSM) of nanocomposites under low velocity punch-shear loading.

In the case of low velocity punch-shear, the correspondence between surface fractal dimensionality and nanoreinforcement is somewhat decreasing (Figure 11) except 1.25 wt % xGnP reinforced nanocomposite which is indicating that the fracture surface for this nanocomposite configuration was the most tortuous. The fractal dimension decreases, however, when this reinforcement is supplemented with 10 wt % CTBN toughening agent.

**Figure 12:**
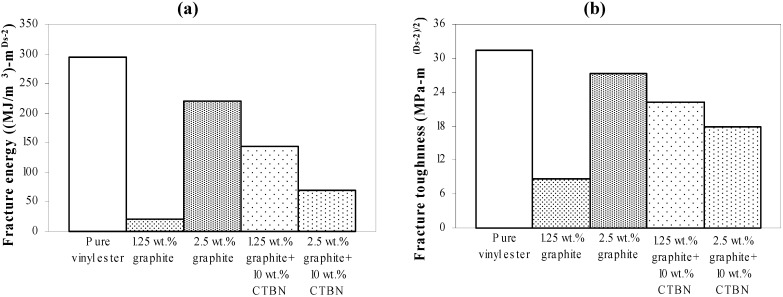
Theoretically estimated (**a**) fracture energy; and (**b**) fracture toughness of nanocomposites under low velocity punch-shear.

Figure 12a compares the nanoreinforced fracture energy and toughness estimated theoretically using Equations 3 to 5 for each material configuration. It reflects a similar decreasing trend with respect to the experimentally obtained fractal dimension shown in Figure 11, except for the 1.25 wt % graphite reinforcement. Figure 12b illustrates the fracture toughness of these nanocomposites estimated from Equation 5, and shows the same trend as fracture energy (Figure 12a).

**Figure 13:**
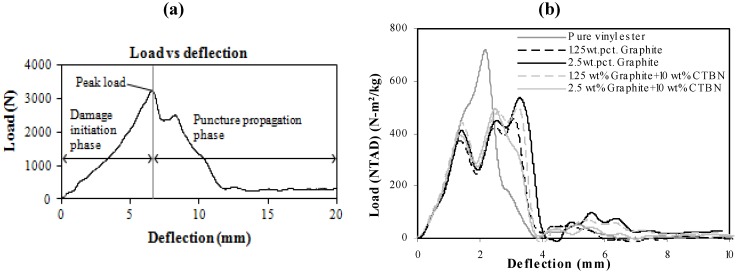
(**a**) Damage initiation and puncture propagation phase on a typical load-deflection response of nanocomposites under low velocity punch-shear; and (**b**) Experimentally obtained load (NTAD)-deflection response [17].

**Figure 14:**
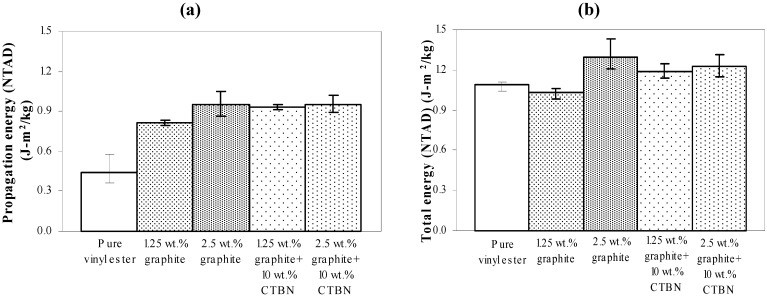
Experimentally obtained (**a**) Energy (NTAD) absorption during puncture propagation, and (**b**) total energy (NTAD) absorption of nanocomposites under low-velocity impact.

In low velocity punch-shear tests, damage propagation due to puncture is observed to occur after the first peak load of the load-deflection response. Hence, as shown in Figure 13a, and also described in Figure 1, the total load-deflection response is divided into two phases, *i.e.*, damage initiation phase and damage propagation phase. The load-deflection response (Figure 13b) from low velocity punch-shear tests on the same set of nanocomposites was studied in a previous investigation [17]. The amount of energy absorbed during each phase is the area under the corresponding portion of the load-deflection curve. Fracture energy is only a part of the total energy absorbed during the puncture propagation phase. In the post-test fractured specimens investigated here, the theoretically estimated fracture energy (shown in Figure 12a) does not show a trend similar to either energy absorption due to damage propagation (Figure 14a), or the total energy absorption (Figure 14b). 

**Figure 15:**
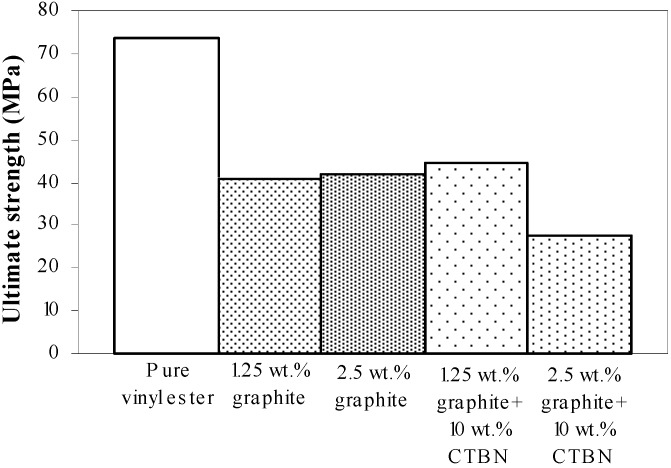
Ultimate strength of vinyl ester nanocomposites from quasi-static axial tensile tests.

Figure 15 shows the ultimate strength of these graphite platelet reinforced nanocomposites obtained experimentally from quasi-static uniaxial tensile tests. It is observed that the ultimate strength is decreasing with increasing nanoreinforcement, similar to the theoretically estimated fracture energy (shown in Figure 9a and Figure 12a), for both quasi-static and punch-shear failed specimens. The surface fractal dimensionality (shown in Figure 8) increases with decreasing fracture energy and toughness, for quasi-static tensile (Figure 9) loading. This trend is consistent with the ductile response reported in literature [12], and as shown on the right side of Figure 2. On the other hand, low velocity punch-shear tested specimens illustrate decreasing trend for both surface dimensionality (Figure 11) and the fracture energy (Figure 12), which is in agreement with the brittle response shown on the left side of Figure 2. These two different trends between the fracture energy and surface fractal dimensionality for quasi-static and punch-shear loading are conceivable for composite materials [12]. 

## 4. Conclusions 

Applicability of fractal analysis to the study of fractured surfaces in nanoparticle reinforced composites has been investigated. During crack propagation, tortuosity varies at different scales, due to which the fractured surface becomes self-affine at the nano, micro and macro-levels, whereas the currently accepted methodologies are based on self-similar fractals. Hence, dimensionality varies with measuring scales and/or magnification factors and different definitions of dimensionality as well. Determining an invariant fractal dimensionality of the surface becomes dependent on sampling at multiple scales and thus, more tedious. 

In the case of nanoparticle reinforced composite materials, heterogeneity of the fracture surface morphology at multiple scales dictates the possible uncertainty in determining an overall fractal dimensionality. In Vertical Section Method, which is based on surface fractal measurements covering a broad range of scales with limited steps of magnification, the possibility for systematic errors is higher. In the case of Slit Island Method, however, since the fractal dimensionality is estimated at a certain scale, such errors are reduced but at the expense of introducing scale dependent estimates. In the work reported here the Vertical Section Method has been used.

In the work reported here, it is observed that the fracture energy and toughness are highly sensitive to fractal dimensionality. The fractal dimensionality increases with wt % increase in xGnP reinforcement under quasi-static uniaxial tensile loading, whereas low velocity punch-shear tested specimens show decreasing trend. An anomaly is observed, however, with 1.25 wt % xGnP which shows the highest dimensionality in case of low velocity punch-shear. The estimated fracture energy and toughness decrease with nanoreinforcement increment for the specimens failed in both quasi-static tensile and low velocity punch-shear loading. This decreasing trend is similar with that of the ultimate strength obtained from quasi-static tensile test. For the graphite platelet reinforced nanocomposites investigated, surface fractal analysis has depicted the probable ductile or brittle fracture propagation mechanism, depending upon the rate of loading. 
